# Accelerating polygon beam with peculiar features

**DOI:** 10.1038/s41598-018-26737-2

**Published:** 2018-06-05

**Authors:** Zhao-Xiang Fang, Hong-Ze Zhao, Yue Chen, Rong-De Lu, Li-Qun He, Pei Wang

**Affiliations:** 1 Physics Experiment Teaching Center, University of Science and Technology of China, Hefei, 230026 China; 2 Department of Optics and Optical Engineering, University of Science and Technology of China, Hefei, 230026 China; 3 Caltech Optical Imaging Laboratory, Andrew and Peggy Cherng Department of Medical Engineering, Department of Electrical Engineering, California Institute of Technology, Pasadena, California, 91125 USA; 4 Department of Modern Physics, University of Science and Technology of China, Hefei, 230026 China; 5 Department of Thermal Science and Energy Engineering, University of Science and Technology of China, Hefei, 230026 China

**Keywords:** Optics and photonics, Optical manipulation and tweezers

## Abstract

We report on a novel kind of accelerating beams that follow parabolic paths in free space. In fact, this accelerating peculiar polygon beam (APPB) is induced by the spectral phase symmetrization of the regular polygon beam (RPB) with five intensity peaks, and it preserves a peculiar symmetric structure during propagation. Specially, such beam not only exhibits autofocusing property, but also possesses two types of accelerating intensity maxima, i.e., the cusp and spot-like structure, which does not exist in the previously reported accelerating beams with a single kind of lobes. We also provide a detailed insight into the theoretical origin and characteristics of this spatially accelerating beam through catastrophe theory. Moreover, an experimental scheme based on a digital micromirror device (DMD) with the binary spectral hologram is proposed to generate the target beam by precise modulation, and a longitudinal needle-like focus is observed around the focal region. The experimental results confirm the peculiar features presented in the theoretical findings. Further, the APPB is verified to exhibit self-healing property during propagation with either obstructed cusp or spot reconstructing after a certain distance. Hence, we believe that the APPB will facilitate the applications in the areas of particle manipulation, material processing and optofludics.

## Introduction

As a special type of structured lights, spatially self-accelerating beams have attracted much attention in recent years. The interest in this kind of beams was aroused when a non-spreading Airy wavepacket initially proposed in the context of quantum mechanics^1^, was introduced into the optical domain^2,3^. Besides maintaining an invariant lateral intensity profile, the Airy beam moves along parabolic trajectory in free space, betraying our common sense that light beams propagate along straight paths. With this peculiar property, such beam can benefit a variety of applications that ranges from the areas of microscopy^4^, spatiotemporal light bullets^5^, light-induced curved plasma channels^6,7^ to particle manipulations^8^. Interestingly, the Airy mode was also realized in nonlinear media^9^ and in other forms of waves, such as self-bending electron beams^10^, acoustic waves^11^ and surface water waves^12^. In addition to the classical Airy beams, the generalized Airy beams and their properties were recently studied^13,14^. Specially, another kind of accelerating parabolic beam that also fulfills the exact solution of two-dimensional (2D) paraxial wave equation was proposed^15^. Such beam shares the same diffraction-free property as the Airy beam, but has an inherent parabolic shape, and its propagation dynamics were also experimentally demonstrated^16^. However, these types of paraxial accelerating beams are limited by their paraxial essence, thus can not preserve their shape-invariant feature when bending to larger angles. Afterwards, research efforts have been devoted to overcome such limitation, and the paraxial accelerating beams have been extended to the nonparaxial regime. In the past few years, nonparaxial accelerating beams (NABs), for instance, Mathieu and Weber beams that were found as exact solution of the Helmholtz equation, have been proposed and demonstrated experimentally^17^. Further, the methods explored to tailor these beams capable of following arbitrary convex^18^ or predesigned paths were investigated^19,20^. Other kinds of accelerating beams, such as accelerating Bessel-like beams^21^, non-diffracting spatially accelerating beams^22^ and incoherent self-accelerating beams were also proposed^23^. Those NABs and other types of accelerating beams may provide larger flexibility of modulating the desired beam trajectories in practical applications.

Recently, another family of finite-energy accelerating regular polygon beams (RPBs) was predicted in theory^24^. Such beams possess an odd number of accelerating high-intensity peaks that accelerate along curved trajectories during propagation. Besides, the intensity maxima resemble a cusp point shape and are equally situated along a circle whose center is the optical axis. Since then, related researches have been induced. The accelerating triple or quinary-cusp beams were experimentally realized and their propagation dynamics in free space were investigated^25,26^. On the other hand, accelerating polygon beams with even-number intensity peaks that exhibit symmetry and autofocusing property have been predicted and experimentally observed recently^27^. Although there is a close relationship between the properties of acceleration and autofocusing^28,29^, accelerating beams constructed in a certain way may not have autofocusing properties^30^. Those experimentally generated accelerating cusp beams were realized by shaping their phase distribution in the spectrum space with a liquid-crystal spatial light modulator (LC-SLM). Actually, self-accelerating beams can be perceived as a caustic wave phenomenon based on diffraction catastrophe theory. For instance, the Airy beams are examples of a canonical catastrophe, i.e., the hyperbolic umblics (HUs), whereas the RPBs belong to the elliptic umblics (EUs)^24^. Both HUs and EUs are separate classes for real variables but merge into one for complex transformation. Furthermore, in terms of the phase distribution and optics characteristics, the relations between the Airy beams and accelerating triple-cusp beams have been discussed^31^.

Here, we present a new class of accelerating beams both in theory and experiment, noted as accelerating peculiar polygon beam (APPB), whose profile exhibits transversely rectangular symmetry and looks like four spots space equally along a circle that are surrounded by four accelerating cusp points situating on the corners of a square. As a matter of fact, it arises from the accelerating quinary-cusp beam by only changing the odd parity to the even parity in its spectral phase. Different from the reported RGBs with only one single kind of accelerating cusp high-intensity peak, such beam has two various types of accelerating lobes, which are referred to as the cusp and spot peaks that exist in the vertices of the polygon structure. On the other hand, it simultaneously shares peculiar features of autofocusing and self-accelerating during propagation. In order to verify these findings, the creation of APPB is implemented through a digital micromirror device (DMD). The advantage of DMD is its capability to present the diffractive patterns with a considerably fast speed^32^. Entire modulation over the complex amplitude modulation is achieved through binary amplitude holograms designed by a super-pixel algorithm^33^. Consequently, the experimentally generated intensity profiles demonstrate the beam propagation property in free space and the needle-like shape around the focal region that caused by autofocusing behavior can be observed. Further, the created APPB is verified to exhibit self-healing property during propagation with either obstructed cusp or spot point reconstructing after a certain distance. Notably, such unique kind of structured beam with two types of intensity maxima can offer great potentials in the practical applications. Thus, we anticipate this novel kind of accelerating polygon beam will benefit the applications in the areas of particle manipulation, precise material processing and optofluidics.

## Results

### Principle and numerical simulation

Based on the relation between the diffraction integrals and catastrophe polynomials, the three-dimensional (3D) complex amplitude distribution of accelerating regular polygon beams can be derived, and it is in the form of integral representation^24,25^.

1 $$E(x,y,z)=A{\int }_{-\infty }^{\infty }{\int }_{-\infty }^{\infty }exp[ik\phi (\varepsilon ,\eta ,x,y,z)]d\varepsilon d\eta $$

where *φ*(*ε*, *η*, *x*, *y*, *z*) = *ϕ*(*ε*, *η*) − *u*(*ε*^2^ + *η*^2^) − (*xε* + *yη*) denotes the canonical polynomial that is mainly determined by the beam phase function *ϕ*(*ε*, *η*), whereas (*x*, *y*) represent the transversal coordinates and *z* is the longitudinal coordinate in the spatial domain, (*ε*, *η*) are the corresponding spectral variables, *u* is the defocus strength *z*/2*f*(*f* + *z*), *z* is the distance from the focal plane along the optical axis, *k* = 2*π*/*λ* is the wavenumber and *A* is a constant associated with the amplitude scale.

The phase function for accelerating polygon beam with five cusp points in the spectral space is expressed as:

2 $$\varphi (\varepsilon ,\eta )={\varepsilon }^{5}+{\eta }^{5}+5({\varepsilon }^{4}\eta +\varepsilon {\eta }^{4})-10({\varepsilon }^{3}{\eta }^{2}+{\varepsilon }^{2}{\eta }^{3})$$

This kind of accelerating quinary-cusp beam was theoretically predicted in 2010 ^24^. Recently, such beam has been experimentally demonstrated by projecting the predesigned spectral phase mask onto the LC-SLM, and the beam profiles can be observed at the focal plane of a Fourier transforming lens^31^. Here, we propose a novel kind of APPB by only changing the odd to even parity in Eq. (2). Thereby, its spectral phase expression is described as:

3 $$\varphi (\varepsilon ,\eta )={|\varepsilon |}^{5}+{|\eta |}^{5}+5({|\varepsilon |}^{4}|\eta |+|\varepsilon |{|\eta |}^{4})-10({|\varepsilon |}^{3}{|\eta |}^{2}+{|\varepsilon |}^{2}{|\eta |}^{3})$$

By associating Eqs (1) and (3), we can obtain the intensity distributions of such APPB propagating in free space. Besides, in order to facilitate the numerical simulation, the dimensionless variables and parameters are adopted, where *s*_*x*_ = *x*/*x*_0_ and *s*_*y*_ = *y*/*y*_0_ are the dimensionless transverse coordinates; *x*_0_ and *y*_0_ are the length scales, and $\xi =u/k{x}_{0}^{2}$ is a normalized propagation distance.

**Figure 1:**
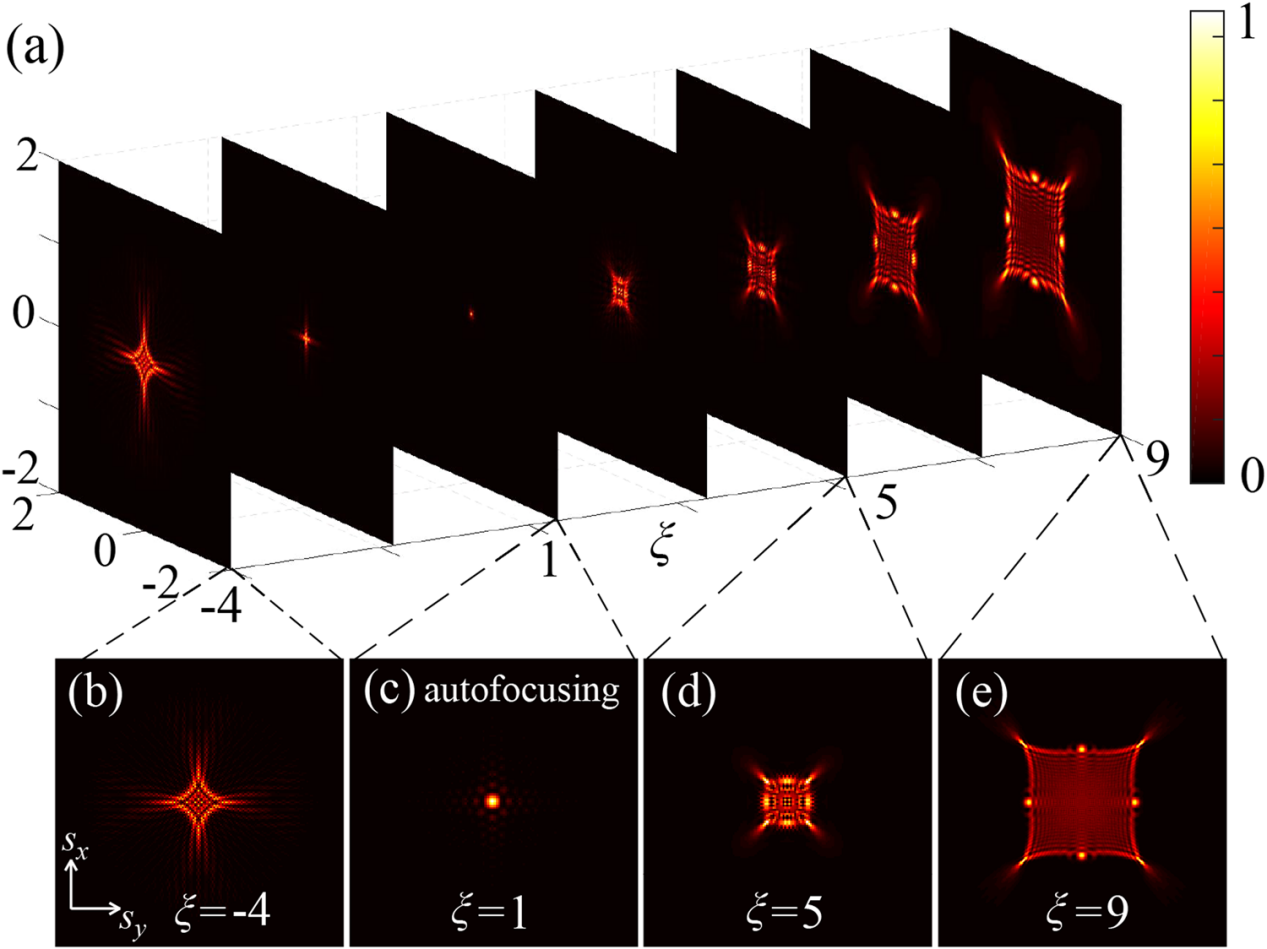
Transversal beam profiles for the APPB at various propagation distance. (**a**) Seven cross-sectional profiles of various planes in free space. (a1)–(a4) represent the transversal intensity profiles that correspond to the certain planes shown in the (**a**), and each position marked by two dashed lines.

The Fig. 1 numerically shows the intensity profiles for the APPB propagating at various transverse planes and corresponding sliced patterns. In simulation, the overall propagation range includes the negative values of *ξ* that corresponds to the positions in the negative direction, considering the distance between the focal plane and autofocusing position is normalized to 1. Note that, we also normalize the intensity distribution to the maximum intensity at each plane, instead of normalizing it to the intensity at autofocus. Therefore, it can benefit the visualization of beam structure on each transverse plane.

Initially, at the position of negative *ξ* the optical shape appears with symmetric cusp caustics that exhibits the diamond-like intensity distribution, which is shown in (a1). Then, the beam focuses into a single lobe around the focal plane and such special autofocusing behavior maintains until it reaches the extreme intensity value at *ξ* = 1 depicted in (a2). After the autofocus, the central lobe collapses and gradually diffuses into an intriguing beam contour with eight intensity maxima placed equally in a symmetric but peculiar polygon shape. Interestingly, as opposed to the reported accelerating quinary-cusp beams^31^, the APPBs not only possess symmetric transverse profiles together with features of autofocusing and self-accelerating, but also have another unique kind of spot-like peak intensity distribution. Specifically, the multiple high-intensity peaks that follow curved paths in free space are classified into two various types of intensity maxima points, which are referred to as accelerating cusp and spot high-intensity peaks. The cusp points are spaced at four corners of a square, and each spot apex lies in the center of the concave side. The peak intensity of cusp is higher than that of spot, and the ratio between two lobes is 1.2.

From the perspective of catastrophe theory, the phase of angular spectrum obtained from the catastrophe integral determines the geometric structure of a caustic beam and its propagation dynamics^34^. Specifically, the symmetric phase yields a symmetric intensity distribution with respect to the propagation axis *ξ*. Further, the generating function *φ*(*ε*, *η*, *x*, *y*, *z*) is introduced to analyze the caustic beam structure of the entire propagation range. From the Eq. (1), this function is constituted by three terms, and as the first term, the spectral phase is the origin of the generating function, whereas the other two terms represent the paraxial propagator term and the Fourier transform term respectively. Once ∇*φ* = 0 and *det*{*H*(*φ*)} = 0, the singularity set will be defined, and it is a subset of the catastrophe manifold, which is the set of all critical points, where ∇ is the gradient with respect to *ξ* or *η*, and *det* denotes the determinant and *H*(*F*) represents the Hessian matrix defined by a square matrix of second-order partial derivatives of *φ*.

Note that, the projection S onto the (*x*, *y*, *u*) is the bifurcation set *B*, which gives the envelope of the highest intensity areas known as caustics in spatial domain^24,35^. In our 2D case, the elements of *S* satisfy:

4 $$\{\begin{array}{c}\frac{\partial \varphi }{\partial \varepsilon }-2u\varepsilon -x=0\\ \frac{\partial \varphi }{\partial \eta }-2u\eta -y=0\end{array}$$

5 $$det[\begin{array}{cc}\frac{{\partial }^{2}\varphi }{\partial {\varepsilon }^{2}}-2u & \frac{{\partial }^{2}\varphi }{\partial \varepsilon \partial \eta }\\ \frac{{\partial }^{2}\varphi }{\partial \varepsilon \partial \eta } & \frac{{\partial }^{2}\varphi }{\partial {\eta }^{2}}\end{array}]=0$$

Substituting Eq. (3) into the Eqs (4) and (5), we can deduce such following equations:

6 $$\{\begin{array}{l}5sgn(\varepsilon ){|\varepsilon |}^{4}+\mathop{\sum }\limits_{i\mathrm{=1}}^{4}i{c}_{i}\,\ast \,sgn(\varepsilon )\,\ast \,{|\varepsilon |}^{i-1}{|\eta |}^{5-i}-2u\varepsilon -x=0\\ 5sgn(\eta ){|\eta |}^{4}+\mathop{\sum }\limits_{i\mathrm{=1}}^{4}(n-i){c}_{i}\,\ast \,sgn(\eta )\,\ast \,{|\varepsilon |}^{i}{|\eta |}^{5-i-1}-2u\eta -y=0\\ 200{({\varepsilon }^{2}+{\eta }^{2})}^{3}-{u}^{2}=0\end{array}$$

**Figure 2:**
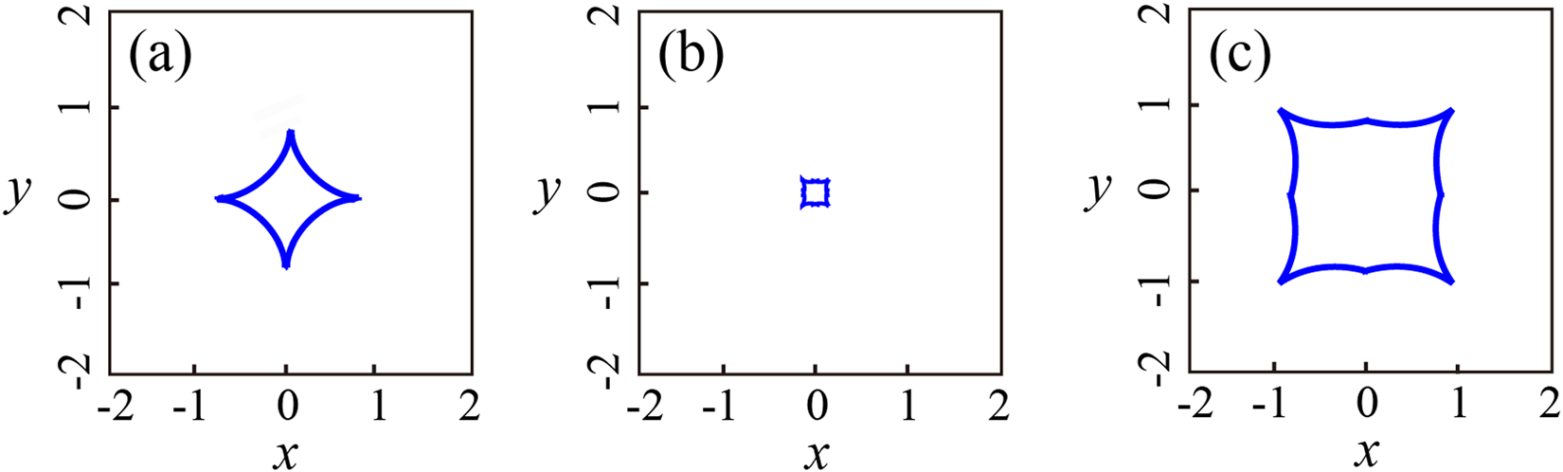
The calculated beam envelopes with various propagation distance (**a**) *ξ* = −5, (**b**) *ξ* = 1 and (**c**) *ξ* = 10.

The variable *c*_*i*_ = (5, −10, −10, 5) and *sgn* represents the sign function, and it is introduced to facilitate the differentiating calculation^34,36^. In Eq. (6), by modulating the parameter *u* and eliminating spectral variables (*ξ* or *η*), we can obtain the calculated beam envelopes with various propagation distances (Fig. 2), which agree well with the numerical intensity distribution as presented in Fig. 1.

### Beam generation with a binary DMD

In order to experimentally implement our findings, we exploit the setup that based on the DMD and super-pixel method for generation of APPBs (see Methods). Compared with the common LC-SLM, DMD is a better candidate for tailoring optical waves due to its fast switchable and high fill factor merits, and it requires a binary amplitude hologram related with target beam^37,38^, although there is a tradeoff between energy losses and the accuracy of beam information^39^. To enable the precise phase modulation of the desired beam, the projected binary pattern is numerically designed by the super-pixel method to create the target hologram^33^.

Widely used in the digital light processing technology, the DMD (DLP 7000, XGA, Texas Instrument) is composed by an array of microscopic mirrors that can be individually rotated into two angular conditions and each of them leads to an on-off modulation of the reflected output beam at that specific pixel position. This results in a binary amplitude modulation of the incident light beams according to the micromirror state. As the DMD can be considered as a binary SLM, binary amplitude masks are designed to accurately encode complex amplitude information based on the super-pixel method. The combination of this method and amplitude DMD can realize a high modulation fidelity and great robustness in the generation of various spatial beam modes.

**Figure 3:**
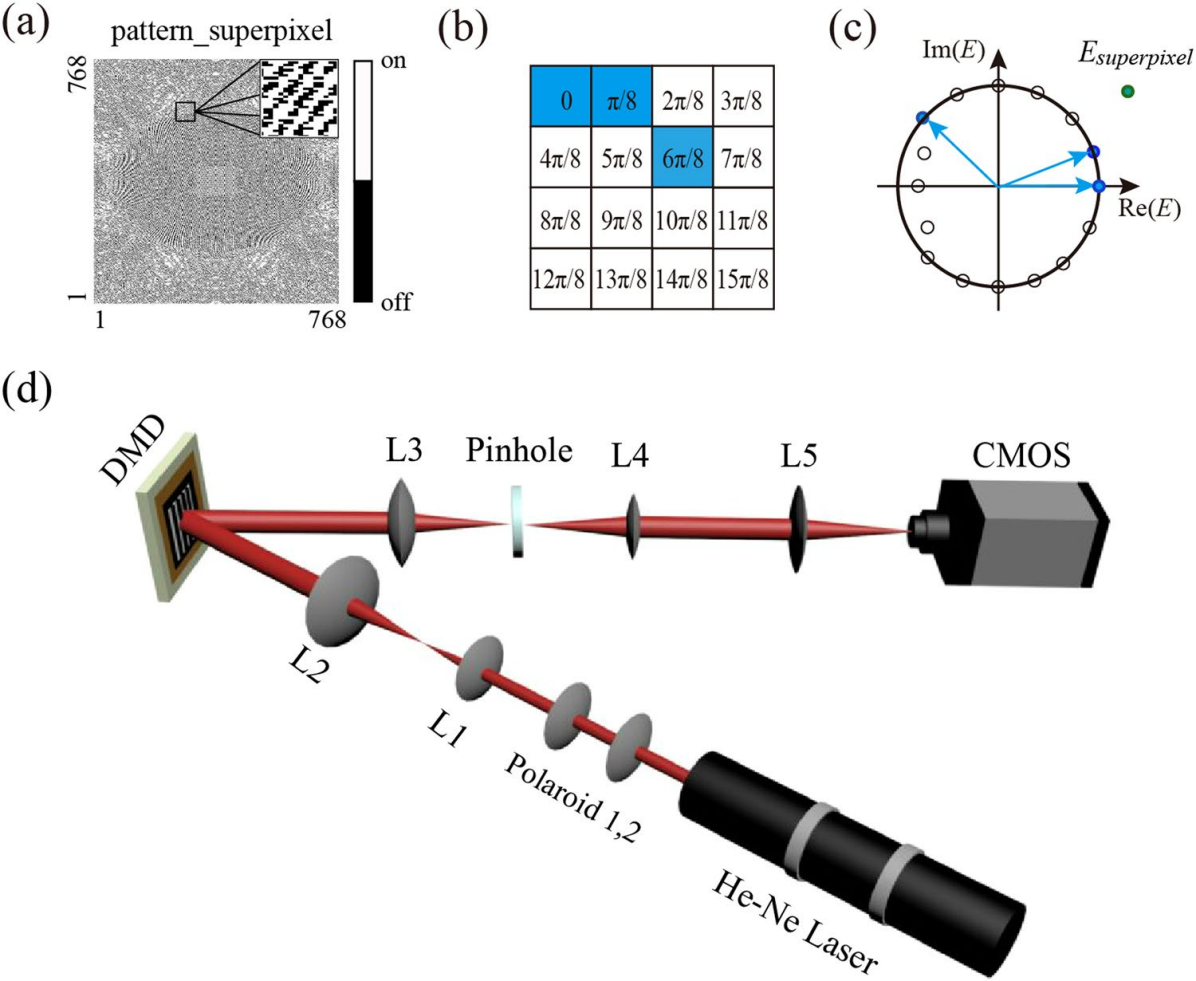
Schematic diagram for generating the APPB. (**a**) The binary hologram for the creation of the target beam. (**b**) Principle of a giant pixel composed of 4 × 4 micromirrors, and the number in each square denotes the phase difference with respect to the corner pixel. (**c**) The response of superpixel in the target plane is the sum of the three sub-pixel responses. (**d**) The experimental setup for the creation of the target beam.

The experimental setup for generating the APPB is presented in Fig. 3(d). A single mode He-Ne laser (Throlabs) is employed as the coherent light source. In order to fill the surface of the DMD, the coherent beam is expanded and then collimated through a telescope composed of dual lenses, whose focal lengths of lens L1 and L2 are 25 mm and 200 mm respectively. Lens L3 (200 mm) acts as a Fourier-transforming lens that collects the tailored light to the spatial space. After L3, undesired optical signals are blocked by a spatial filter that locate at the back focal plane of L3, and the desired signal is imaged by an additional 4 f setup, which consists of lens L4 (100 mm) and L5 (200 mm). In Fig. 3(d), *z* = 0 represents the focal plane of the L5, and moving away from the focal plane corresponds to different propagation distance *z*. Finally, a movable CMOS camera (Weiyu Cooperation) allows us to the capture the target beam profiles in the propagation direction. With the proposed setup, we are able to cover almost the entire parameter range provided by our theory.

**Figure 4:**
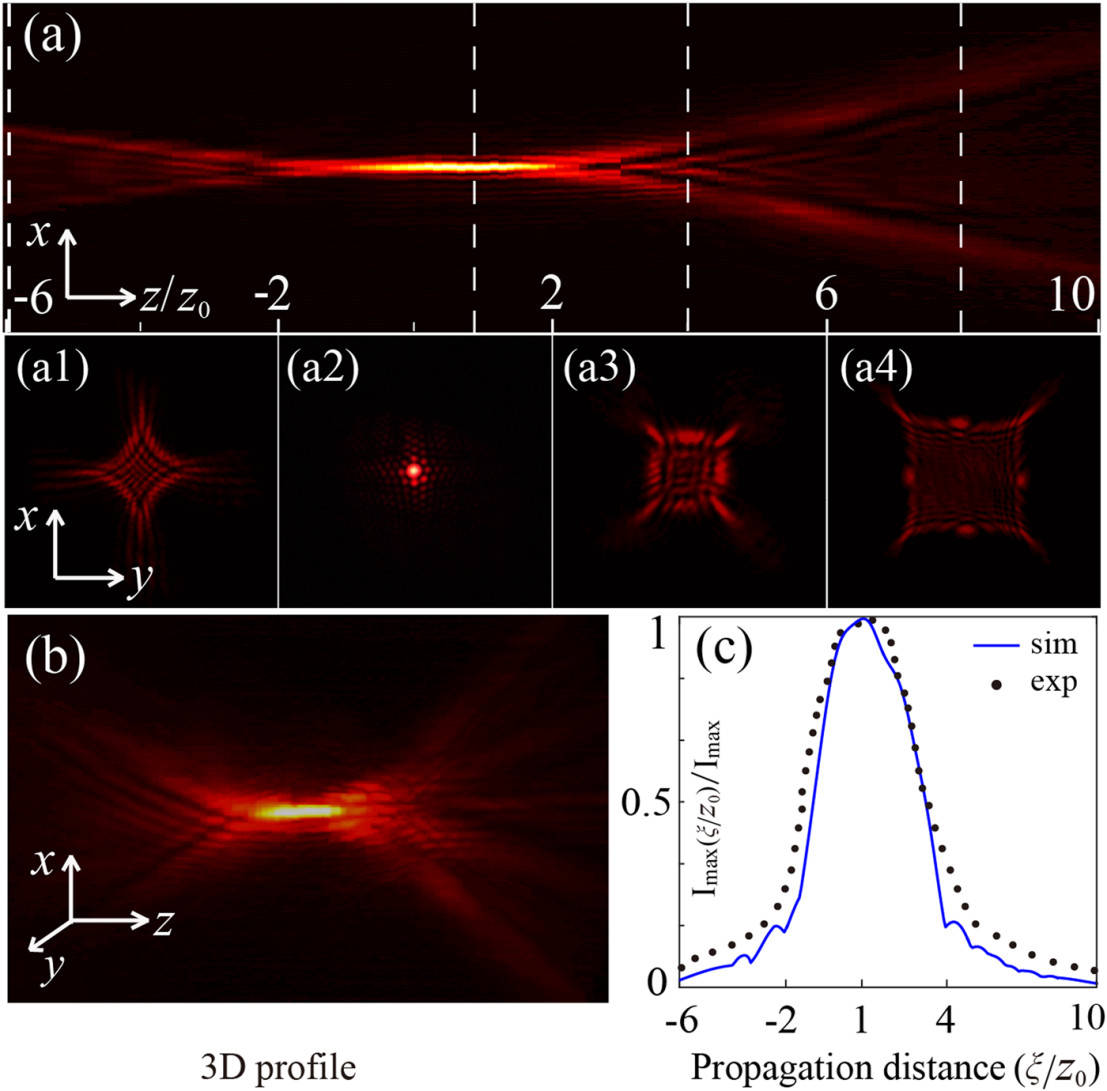
Experimental demonstration of the APPB propagating in free space. (**a**) side-view beam profile of the APPB; (a1)–(a4) represent transverse beam profiles at the planes *z* = −6*z*_0_, *z*_0_, 4*z*_0_ and 8*z*_0_ (*z*_0_ = 0.8 cm), marked by the dashed lines in (**a**), respectively; (**b**) the 3D view of the beam field taken from a certain angle of view. The peak intensity distribution as a function of propagation distance is shown in (**c**).

The propagation dynamics of the APPB is experimentally verified and demonstrated in Fig. 4. Here, the propagation distance is normalized to *z*_0_ that represents the distance between the autofocusing position and the back focal plane of the focusing lens L5, where *z* = 0 corresponds to the L5 focal plane. The intensity distribution at each transverse plane is normalized to the maximum intensity at autofocus, so that the intensity distribution of transversal patterns coincide with that of the side-view profile in various propagation distances, displayed in Fig. 4(a) and (a1)–(a4).

### Self-healing property of two various lobes

**Figure 5:**
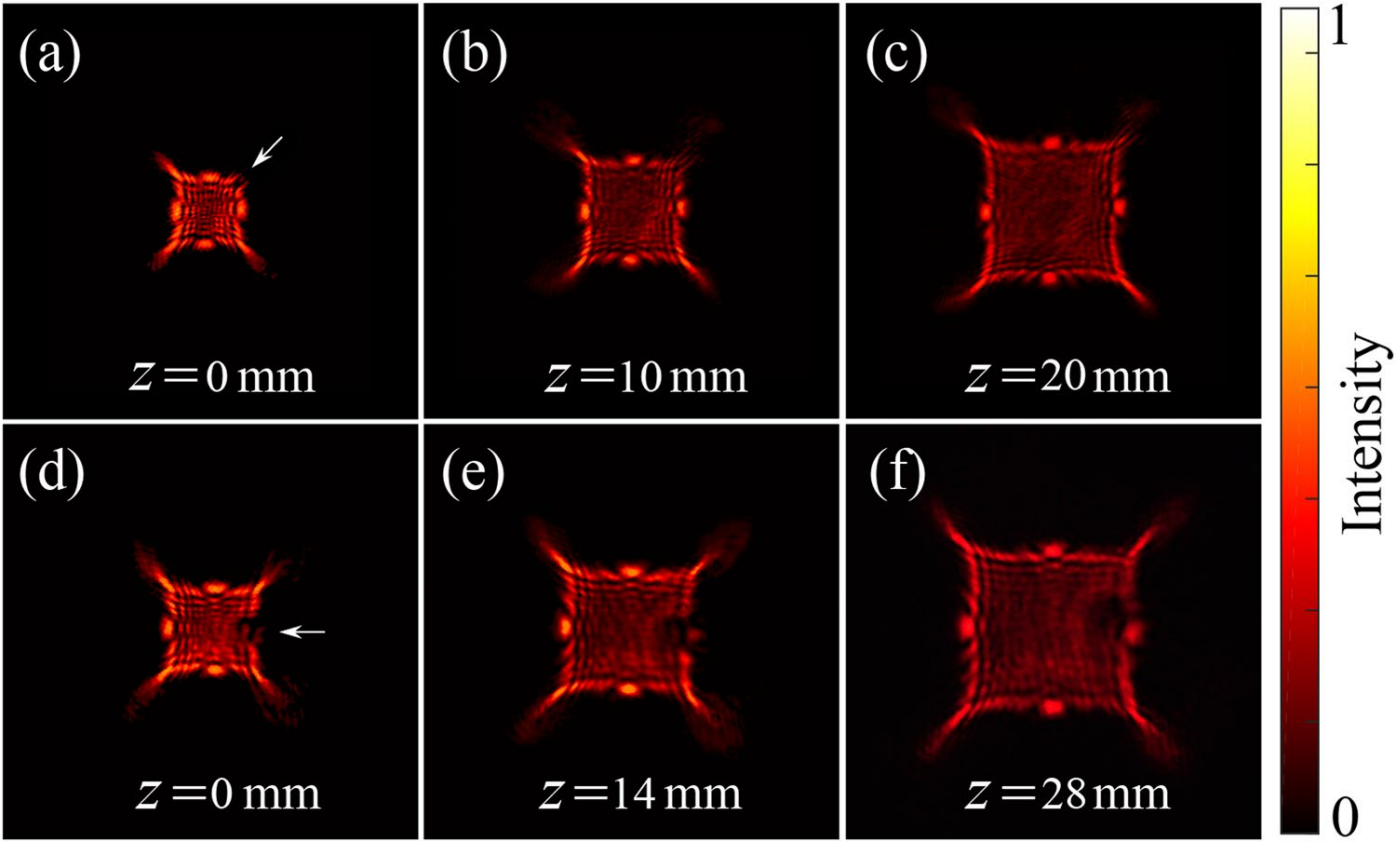
Experimental results for self-healing behavior of the generated APPB. The beam profile with one of cusp points blocked locates at (**a**) 0 mm, (**b**) 10 mm, and (**c**) 20 mm; Self-healing results for the beam with one of the spot points blocked (second row), and the recovered intensity profiles are observed at (**d**) 0 mm, (**e**) 14 mm and (**f**) 28 mm behind the obstacle and the white arrow points to the blocked area.

Finally, the optical stability of the APPBs are experimentally investigated to verify whether such beams still possess the same self-healing property as presented in the accelerating quinary-cusp beams. In Fig. 5, a circular opaque mask is adopted to obstruct the cusp point at the top-right region, and the initial position is set as z = 0 mm (see Methods). At the original plane shown in Fig. 5(a), the cusp immediately disappeared for the occurrence of the obstacle, then the blocked cusp point gradually reconstructs at z = 10 mm (Fig. 5(b)), and it basically recovered itself with the entire shape of the cusp point reappeared at position z = 20 mm, displayed in Fig. 5(c). Moreover, we investigate if such self-recovering feature also exists in another type of accelerating lobes, i.e., the spot maxima points. Later, this kind of intensity peaks was verified to exhibit the self-healing property during propagation with the obstructed right spot point reconstructing through the observation of experimental measurements. From the second row of Fig. 5, it took a little longer distance for the spot point to realize self-recovering process. These experimental results manifest that such APPB, derived from the symmetrization for the spectral phase of the reported accelerating quinary cusp beam, still maintains self-healing feature. Further, such kind of beam with two type of accelerating lobes both sharing this self-recovering behavior can be desirable for light propagation through the atmospheric turbulence or other complex scattering media.

## Discussion

In summary, we have proposed and demonstrated a new class of self-accelerating waves. Theoretically, such APPBs that accelerate freely along parabolic trajectories, were induced by symmetrizing the spectral phase of accelerating quinary cusp beams. Notably, compared with RPBs, such beams not only exhibit autofocusing property, but also possess another kind of accelerating spot-like intensity maxima besides the cusp-points distribution. As a proof of concept, the experimental generation of APPBs from spectrum modulation was presented through employing binary amplitude holograms projected on DMD. With the proposed experimental setup, the scheme of DMD and super-pixel method that accurately encode the target field information is shown to be possible with great flexibility. The measured beam profiles confirm the peculiar beam features proposed in the theoretical analysis, and the longitudinal needle-like structure caused by the autofocusing behavior can be observed around the focal region. Besides, such beam is verified to still exhibit self-healing property during propagation with either obstructed cusp or spot maxima point recovering itself after a certain distance. Thus, we anticipate that such APPBs with intriguing properties will provide potentials in the applications of particle manipulation, material processing and optofludics.

## Methods

### Full beam field modulation employing a binary amplitude hologram

For the experimental scheme, a DMD is exploited for the target wavefront shaping. As the DMD is a binary SLM, binary amplitude holograms are designed based on a super-pixel method to accurately encode the entire beam field information^33^. Usually, the Lee method is utilized to generate the holograms for the target beam. As compared with Lee method that only takes one dimension to encode the amplitude and phase in fringes^40^, the super-pixel method provides more precise modulation by adopting both dimensions of pixel arrays.

Schematically, we take Fig. 3(b) and (c) to depict the principle of the super-pixel method^33^. The *n* × *n* micromirrors constitute a super pixel that defines a complex field, and the phase prefactors caused by the target field responses of the micromirrors with each super pixel distributes uniformly between 0 and 1. In this case, we divide the entire micro-mirrors into 4 × 4 superpixels. The target responses of individual pixel (dark dot) in a superpixel are uniformly distributed on a circle with a phase step of *π*/8. Once only the blue dots are switched on (active), the synthesized response of the superpixel (green dot) can be obtained by superposition of the entire active pixel responses. With this method, the binary hologram can be obtained, shown in (a). Then, the APPB can be obtained with the binary amplitude hologram projected onto the DMD.

### Material preparation for the self-healing experiment

We utilized an ink dot tagged on a glass slide with diameter of about 200 microns as an opaque obstacle, which was placed directly in the beam propagation path to block the spot or cusp point area. Then, the self-healing behavior was confirmed by observing the target beam intensity distribution behind the obstruction.
