# Author Correction: Accelerating polygon beam with peculiar features

**DOI:** 10.1038/s41598-019-54457-8

**Published:** 2019-11-25

**Authors:** Zhao-Xiang Fang, Hong-Ze Zhao, Yue Chen, Rong-De Lu, Li-Qun He, Pei Wang

**Affiliations:** 1 Physics Experiment Teaching Center, University of Science and Technology of China, Hefei, 230026 China; 2 Department of Optics and Optical Engineering, University of Science and Technology of China, Hefei, 230026 China; 3 Caltech Optical Imaging Laboratory, Andrew and Peggy Cherng Department of Medical Engineering, Department of Electrical Engineering, California Institute of Technology, Pasadena, California, 91125 USA; 4 Department of Modern Physics, University of Science and Technology of China, Hefei, 230026 China; 5 Department of Thermal Science and Energy Engineering, University of Science and Technology of China, Hefei, 230026 China

Correction to: *Scientific Reports* 10.1038/s41598-018-26737-2, published online 05 June 2018

The original version of this Article contained errors in the Abstract.

“We report on a novel kind of accelerating beams that follow parabolic paths in free space. In fact, this accelerating peculiar polygon beam (APPB) is induced by the spectral phase symmetrization of the regular polygon beam (RPB) with five intensity beam (RPB) with five intensity peaks, and it preserves a peculiar symmetric structure during propagation. Specially, such beam not only exhibits autofocusing property, but also possesses two types of accelerating intensity maxima, i.e., the cusp and spot-point structure, which does not exist in the previously reported accelerating beams. We also provide a detailed insight into the theoretical origin and characteristics of this spatially accelerating beam through catastrophe theory. Moreover, an experimental scheme based on a digital micromirror device (DMD) with the binary spectral hologram is proposed to generate the target beam by precise modulation, and a longitudinal needle-like focus is observed around the focal region. The experimental results confirm the peculiar features presented in the theoretical findings. Further, the APPB is verified to exhibit self-healing property during propagation with either obstructed cusp or spot intensity maxima point reconstructing after a certain distance. Hence, we believe that the APPB will facilitate the applications in the areas of particle manipulation, material processing and optofludics.”

now reads:

“We report on a novel kind of accelerating beams that follow parabolic paths in free space. In fact, this accelerating peculiar polygon beam (APPB) is induced by the spectral phase symmetrization of the regular polygon beam (RPB) with five intensity peaks, and it preserves a peculiar symmetric structure during propagation. Specially, such beam not only exhibits autofocusing property, but also possesses two types of accelerating intensity maxima, i.e., the cusp and spot-like structure, which does not exist in the previously reported accelerating beams with a single kind of lobes. We also provide a detailed insight into the theoretical origin and characteristics of this spatially accelerating beam through catastrophe theory. Moreover, an experimental scheme based on a digital micromirror device (DMD) with the binary spectral hologram is proposed to generate the target beam by precise modulation, and a longitudinal needle-like focus is observed around the focal region. The experimental results confirm the peculiar features presented in the theoretical findings. Further, the APPB is verified to exhibit self-healing property during propagation with either obstructed cusp or spot reconstructing after a certain distance. Hence, we believe that the APPB will facilitate the applications in the areas of particle manipulation, material processing and optofludics.”

These errors have now been corrected in the PDF and HTML versions of the Article.

